# Assessing the effects of spontaneous intracranial hypotension on quality of life, work ability and disability

**DOI:** 10.1007/s00508-024-02423-4

**Published:** 2024-09-03

**Authors:** Ali Kapan, Thomas Waldhör, Christian Wöber

**Affiliations:** 1 Center for Public Health, Department of Social and Preventive Medicine, Medical University of Vienna, Kinderspitalgasse 15, 1090 Vienna, Austria; 2 Center for Public Health, Department of Epidemiology, Medical University of Vienna, Vienna, Austria; 3 Department of Neurology, Medical University of Vienna, Vienna, Austria; 4 Comprehensive Center for Clinical Neurosciences and Mental Health, Medical University of Vienna, Vienna, Austria

**Keywords:** Spontaneous intracranial hypotension, CSF leak, Headache, Mental well-being, Therapy

## Abstract

**Background:**

Spontaneous intracranial hypotension (SIH), characterized by headaches due to cerebrospinal fluid leaks or low pressure, is a challenging condition to diagnose and treat and affects the quality of life.

**Methods:**

An 8‑week online survey was conducted to assess the impact of SIH on symptoms, sociodemographics and quality of life. The cohort was comprised of patients who had a self-reported diagnosis of SIH and were divided into two groups: those with radiological evidence of SIH and those with clinical suspicion but no radiological evidence. Mental health and disability were evaluated using the Depression, Anxiety and Stress Scale-21 (DASS-21) and the Henry Ford Hospital Headache Disability Inventory (HDI).

**Results:**

A total of 86 participants were included in the study, 59 with radiological evidence and 27 without. Most participants were female (84.9%) with a mean age of 44.8 years. Orthostatic headache was more common in participants without radiological evidence (74.1% vs. 42.4%). The severity in those with radiological evidence was 27.1% mild, 27.1% moderate, 30.5% severe and 15.3% extremely severe, while those without had 7.4% mild, 18.5% moderate, 63.0% severe and 11.1% extremely severe headaches. Mental health assessment using the DASS-21 scale showed that 77.9% of all participants reported signs of depression, 96.5% reported anxiety and 89.5% reported stress. The HDI showed 2.3% total disability, 40.7% severe, 19.8% moderate and 37.2% mild. The impact on employment was significant: 15.1% were able to work full-time, 48.8% part-time, 30.2% were unable to work and 5.8% retired early due to SIH.

**Conclusion:**

The study demonstrates the broad impact of SIH affecting physical health, mental well-being, and socioeconomic status, and calls for multifaceted and robust management approaches to address its complex effects on patients.

## Introduction

Spontaneous intracranial hypotension (SIH), characterized by headaches worsening in an upright position due to cerebrospinal fluid (CSF) leaks or low CSF pressure, presents diagnostic challenges due to its rarity (5 cases per 100,000 person-years) and lack of a definitive test [1]. Patients often experience auditory symptoms like tinnitus or pressure in the ears, sometimes leading to initial misdiagnosis as sudden hearing loss or Meniere’s disease [2]. While the manifestations of SIH can range widely, the condition can significantly affect well-being and lead to long-term complications. In rare instances, it may result in severe reductions in consciousness or even coma [3, 4].

The diagnosis, as outlined by the International Classification of Headache Disorders (ICHD-3), hinges on the onset of the headache in connection with evidence of a CSF leak on imaging or low pressure measured during a spinal tap (lumbar puncture pressure less than 60 mm CSF) [5]; however, it is noted that around two thirds of patients do not meet this criterion as they exhibit a normal opening pressure when supine [6, 7] and that about 20% of individuals display normal brain magnetic resonance imaging (MRI) results [8]. Moreover, insights from a systematic review highlight that various imaging techniques can successfully identify indications of extradural CSF leaks in a limited range of 48–67% of individuals [8]. Epidural blood patches (EBP), the main treatment for unresponsive patients, had a 64% success rate after the first application. Notably, the review also showing that both targeted and non-targeted techniques exhibited comparable efficacy, with larger patches (≥ 20 ml) demonstrating greater effectiveness [8]. Surgical and endovascular repair of CSF leaks and CSF-venous fistulas are promising, yielding significant long-term improvement [9, 10]; however, a recently published study indicated that 1 year post-operation, about one quarter of patients still suffer from headaches that significantly impair their daily lives [11].

Due to the intricate nature of SIH diagnosis and treatment, managing this condition can significantly affect employment, social interactions, health, and overall well-being, as supported by numerous studies [12, 13, 24]. The objective of this study is to gather information on the diagnostic methods and treatment employed, based on patients’ responses.

## Methods

### Settings and participants

This study was conducted as an online survey over a period of 8 weeks from 19 April to 19 June 2023. Ethical approval was obtained from the Ethics Committee of the Medical University of Vienna, under approval number 1180/2023. The online survey was disseminated through social media platforms, including Facebook, Twitter, and Instagram, in collaboration with several organizations. These included organizations, such as spinalcsfleakcanada.ca, csfleak.uk, iatrogenic Lumbar Puncture Leakers, and CSF Leaks International. The publicly accessible online questionnaire was designed in accordance with the Checklist for Reporting Results of Internet E‑Surveys (Appendix, Tab. 7) [14] guidelines using https://www.soscisurvey.de.

As this study is based on patient self-reports and the accurate diagnosis of SIH is complex due to clinical and imaging factors, relatively stringent inclusion criteria were established. Participants in the study had to be at least 18 years old and answer “yes” to all of the following questions:

- Have you been diagnosed with SIH by a physician? If so, we would be grateful if you could complete this questionnaire to the best of your knowledge and belief.
- Have you had persistent headaches for more than 3 months?
- Have you had at least one EBP treatment and at least one MRI scan of your spine and brain?
- Are you able to read and understand English and have you given your consent to take part in the study?

The study was not limited to individuals who reported positional headaches exclusively. This decision was based on recent findings [15] which suggest that the orthostatic nature of these headaches may diminish over time or might not meet the ICHD‑3 criteria (in order for a headache to be classified as orthostatic, it should intensify within 15 min of sitting or standing and alleviate upon lying down). Consequently, although the presence of orthostatic headaches is a useful indicator, the absence does not rule out SIH. Furthermore, individuals diagnosed with postural orthostatic tachycardia syndrome (POTS) and Ehlers-Danlos syndrome (EDS) were included in the study if there was evidence of SIH on imaging. Individuals with a known history of post-dural puncture headache (PDPH) were excluded from the study.

### Survey development

This study was a collaborative effort involving clinical researchers and SIH patients, with a strong emphasis on a patient-centered approach. The survey was divided into 5 sections, ran to 20 pages and contained 106 questions. These sections were:

1. Demographics and characteristics of SIH: collected sociodemographic information, such as age, sex, education and occupation. Sick leave due to SIH, duration of SIH, upright position tolerance, work capacity since onset of SIH.
2. Diagnostics: this section focused on the frequency of medical consultations and detailed the various diagnostic tests that participants had undergone, such as MRI of the brain/spine, CT myelography, CSF pressure measurement, and CSF scintigraphy. Participants were also asked, “What was the final result of all these tests?” Participants could indicate whether the test: 1) confirmed a CSF leak, 2) showed epidural fluid collections, 3) suggested intracranial hypotension (IH) on a brain MRI but showed no abnormalities on a spinal MRI, 4) was inconclusive, or 5) showed no evidence of IH on both brain and spinal MRI.
3. Treatment: To explore the variety and effectiveness of medical treatment for SIH, the duration and associated risks. The questions were designed to be clear and understandable, minimizing the use of complex medical terminology. To assess the effectiveness of treatments, participants were asked: “What symptoms have you had since SIH started?” “How effective has the treatment been in relieving your symptoms in the short term?” and “How effective has the treatment been in relieving your symptoms in the long term?” Participants were asked to rate the effectiveness on a scale from “not effective” to “completely effective”. The terms “short term” and “long term” were deliberately used to allow patients to interpret these time frames in their own way, without the need for further clarification.
4. Impact on personal relationships and emotional health:
  Changes in relationship: participants were asked about changes or difficulties in their personal relationships since the onset of SIH. Responses were categorized as: (1) no noticeable effect, (2) worked through problems, (3) significant difficulties, (4) led to separation/divorce, and (5) preferred not to answer. This allowed us to quantify the extent to which SIH affects personal relationships.
  To assess the emotional impact of SIH, participants were asked if they thought SIH and its consequences had caused any emotional distress. The response options were as follows: (1) yes, I feel a lot of emotional distress, (2) yes, emotionally distressed, but not severely (3) not sure if distressed, (4) no, I have not noticed any negative impact.
5. To assess participants’ general well-being, we utilized standardized evaluation tools, including the Depression, Anxiety and Stress Scale-21 items (DASS-21) [16]. Furthermore, we employed the Henry Ford Hospital Headache Disability Inventory (HDI) [18] to evaluate the severity and frequency of headaches.
  The DASS-21 efficiently measures perceived levels of depression, anxiety, and stress across both clinical and nonclinical adult populations. This brief tool divides its 21 items into 3 scales, with each item rated on a 4-point scale, from 0 (no occurrence) to 3 (very much so). Scores are doubled, reflecting that the tool has a condensed format from the original 42-item DASS. For depression, scores are considered normal up to 9, mild between 10 and 13, moderate from 14 to 20, severe between 21 and 27, and extremely severe at 28 and above. Anxiety scores are normal up to 7, mild at 8–9, moderate from 10 to 14, severe from 15 to 19, and extremely severe at 20 and higher. Stress scores are classified as normal up to 14, mild from 15 to 18, moderate from 19 to 25, severe from 26 to 33, and extremely severe from 34 onwards. This scoring system aids in precisely identifying the severity of each mental health condition, supporting the DASS-21’s broad use in assessing psychological well-being [16, 17].
  The HDI is a well-established instrument widely used to evaluate the severity and frequency of headaches, along with their influence on daily life. It proves particularly effective in assessing no pharmacological interventions’ efficacy among individuals experiencing frequent episodic or chronic migraine, aligning with recommendations from the International Headache Society. Comprising 25 items, the HDI is divided into 2 subscales: a functional subscale comprising 12 items, gauging functional disability, and an emotional subscale consisting of 13 items, evaluating emotional disability. Each item offers 3 response options: ‘no’ (0 points), ‘sometimes’ (2 points), and ‘yes’ (4 points). The total score, representing overall disability, is computed by summing these points, with scores ranging from 0 (indicating no disability) to 100 (indicating maximum disability). The Cronbach alpha reliability coefficients are alpha = 0.76 for the functional subscale, alpha = 0.82 for the emotional subscale, and 0.83 for the total score [18].

### Statistical analysis

The statistical analysis of the study distinguished between two specific groups: the group with radiological evidence included individuals who had a CSF leak, extradural CSF collections or sign of IH on a brain MRI but showed no abnormalities on a spinal MRI. In contrast, the group without radiological evidence included individuals with symptoms suggestive of SIH but without objective findings on radiological imaging. To maintain the integrity of the classification of participants, the statistical analysis included only participants who had received at least one EBP treatment and who had undergone at least one MRI scan of both the spine and the brain. Continuous variables were analyzed and reported as means and standard deviations (SD), while categorical variables were presented as frequencies (*n*) and percentages (%). Normality of data was assessed using the Shapiro-Wilk test and histogram analysis. All statistical evaluations were performed using SPSS® (version 27.0; IBM®, Armonk, NY, USA)  to ensure rigorous and detailed analysis.

## Results

A total of 86 participants were included in the study, 59 with radiological findings and 27 without. The vast majority were female, accounting for approximately 85% of the total sample. The mean age of all participants was 44.8 years and the mean body mass index was 25.4 kg/m^2^. Regarding ethnic background, approximately 90.7% of participants identified as white, while smaller percentages belonged to various other ethnic groups. In terms of marital status, the distribution was as follows: almost 40% were married, one quarter were in a relationship, nearly one third were single, and 8.10% were divorced. Participants exhibited diverse educational backgrounds: About one fifth had completed vocational training or apprenticeships, nearly one fifth were employed, and an additional 17.4% were self-employed. Not quite 6% were university students, while almost 10% were retired. Approximately one third of the participants reported being unable to work due to SIH, further highlighting the impact of this condition on their employment status (Table 1).

**Table 1:** Demographic characteristics of participants

	All participants (*n* = 86)	With radiological evidence (*n* = 59)	Without radiological evidence (*n* = 27)
*Variable*			
**Sex; female**	73 (84.9)	49 (83.1)	24 (88.9)
**Age years mean (SD)**	44 (12.01)	43 (10.9)	42 (11.4)
**BMI (kg/m**^**2**^**) mean (SD)**	25.4 (6.4)	24.6 (5.5)	25.9 (5.9)
**Country, *n* (%)**			
Australia	6 (7.0)	6 (10.2)	0
Austria	2 (2.3)	2 (3.4)	0
Belgium	1 (1.2)	1 (1.7)	0
Canada	11 (12.8)	7 (11.9)	4 (14.8)
Germany	12 (13.9)	9 (15.3)	3 (11.1)
Ireland	1 (1.2)	1 (1.7)	0
Israel	2 (2.3)	1 (1.7)	1 (3.7)
Italy	1 (1.2)	1 (1.7)	1 (3.7)
Norway	1 (1.2)	1 (1.7)	1 (3.7)
Portugal	1 (1.2)	1 (1.7)	1 (3.7)
Spain	5 (5.8)	4 (6.8)	1 (3.7)
Sweden	1 (1.2)	0	1 (3.7)
Switzerland	1 (1.2)	0	1 (3.7)
The Netherlands	1 (1.2)	1 (1.7)	0
Turkey	1 (1.2)	0	1 (3.7)
United Kingdom	10 (11.6)	4 (6.8)	6 (22.2)
USA	25 (29.0)	16 (27.1)	9 (33.3)
**Education, *n* (%)**			
Training/apprenticeship	15 (17.4)	10 (16.9)	5 (18.5)
University student	5 (5.8)	5 (8.5)	0 (0.0)
Employee	15 (17.4)	10 (16.9)	5 (18.5)
Self-employed	15 (17.4)	9 (15.3)	6 (22.2)
Retired	7 (8.1)	7 (11.9)	0
Prefer not to answer	2 (2.3)	1 (1.7)	1 (3.7)
Disabled/unable to work	27 (31.4)	27 (45.8)	10 (30.7)
**Time to SIH diagnosis by a physician, *n* (%)**			
In 24 h	4 (4.7)	3 (5.1)	1 (3.7)
Less than 1 week	5 (5.8)	4 (6.8)	1 (3.7)
1–4 weeks	10 (11.6)	7 (11.9)	3 (11.1)
1–3 months	10 (11.6)	6 (10.2)	4 (14.8)
More than 3 months	57 (66.3)	40 (66.3)	17 (63.0)

*BMI* Body mass Index, *SIH* Spontaneous Intracranial Hypotension, *SD* Standard Deviation, *USA* United States of America

**Table 2:** Characteristics of the SIH in participants with radiological evidence and without radiological evidence

Variable	All participants (*n* = 86)	With radiological evidence (*n* = 59)	Without radiological evidence (*n* = 27)
**Duration SIH, *n* (%)**			
3–6 months	4 (4.7)	3 (5.1)	1 (3.7)
6–12 months	10 (11.6)	7 (11.9)	3 (11.1)
1–3 years	29 (33.7)	20 (33.9)	9 (33.3)
3–5 years	22 (25.6)	15 (25.4)	7 (25.9)
More than 5 years	21 (24.4)	14 (23.7)	7 (25.9)
**Sick leave due to SIH, *n* (%)**			
3–6 months	11 (12.8)	7 (11.9)	4 (14.8)
6–12 months	16 (18.6)	13 (22.1)	3 (11.1)
Over 12 months	23 (26.7)	15 (25.4)	8 (29.6)
Over 2 years	5 (5.8)	4 (6.8)	1 (3.7)
Unable to work since onset of SIH	26 (30.2)	16 (27.1)	10 (37.0)
Retired since onset of SIH	5 (5.8)	4 (6.8)	1 (3.7)
**Work capacity since onset of SIH, *n* (%)**			
Part-time (10–20 h a week)	42 (48.8)	31 (52.5)	11 (40.7)
Full-time (30–40 h a week)	13 (15.1)	12 (20.3)	1 (3.7)
Changed job for lying position	5 (5.8)	2 (3.4)	3 (11.1)
No longer able to work since onset of SIH	26 (30.2)	14 (23.7)	12 (44.4)
**Orthostatic headache, *n* (%)**			
Yes	45 (52.3)	25 (42.4)	20 (74.1)
No	41 (47.7)	34 (57.6)	7 (25.9)
**Upright position tolerance, *n* (%)**			
Not at all	8 (9.3)	5 (8.5)	3 (11.1)
Less than 1 h	36 (41.9)	18 (30.5)	18 (66.7)
2–4 h	7 (8.1)	7 (11.9)	0 (0.0)
Most of the day	7 (8.1)	5 (8.5)	2 (7.4)
Whole day	28 (32.6)	24 (40.7)	4 (14.8)
**Location of headache, *n* (%)**			
Forehead/temples	10 (11.6)	7 (11.9)	3 (11.1)
Whole head	26 (30.2)	18 (30.5)	8 (29.6)
Back of head/neck region	50 (58.1)	34 (57.6)	16 (59.3)
**Severity of headache, *n* (%)**			
Mild	18 (20.9)	16 (27.1)	2 (7.4)
Moderate	21 (24.4)	16 (27.1)	5 (18.5)
Severe	35 (40.7)	18 (30.5)	17 (63.0)
Extremely severe	12 (14.0)	9 (15.3)	3 (11.1)

Those without radiological evidence were more likely to report orthostatic headache (74.1% vs. 42.4%). In terms of headache severity, those with radiological evidence exhibited a distribution of 27.1% mild, 27.1% moderate, 30.5% severe, and 15.3% extremely severe headaches. In contrast, participants without radiological evidence reported 7.4% mild, 18.5% moderate, 63.0% severe and 11.1% extremely severe headaches. In terms of work-related absences due to SIH, approximately one third of all participants reported being unable to work since the onset of the condition, while almost 6% had “retired” due to SIH. A comparable proportion had been absent from work for more than 1 year. It is noteworthy that approximately half of all respondents had been living with SIH for more than 3 years, which serves to illustrate that in many cases it is a chronic condition. In terms of tolerance for an upright position, just over half of all participants were able to stand upright for less than 1h. This factor also influences their ability to work. Approximately half of the participants indicated that they were only able to work part-time, while one third of respondents stated that they were unable to work due to SIH (Table 2).

Table 3 details the diagnostic procedures used, noting that all participants underwent MRI of the brain or spine. CT myelography was also a common procedure, performed in almost 60% of cases. The timing of these examinations varied, with MRIs often performed within the first 3 months of symptom onset, and more than half of CT myelography performed after 6 months. Looking at the number of examinations for MRI, the majority had either 3 or 4, corresponding to 38.4% and 51.2%, respectively. In CT myelography, most patients underwent multiple sessions: 29 out of 51 participants (56.9%) had 2 sessions, 19 individuals (37.3%) completed 3 sessions and 2 participants (3.9%) went through 4 sessions. Further specifics on the frequency and timing of other diagnostic procedures are detailed in Table 3. In terms of diagnostic results, almost one third had confirmed CSF leakage by either CT myelogram or MRI showing either CSF leakage or extradural CSF collection. In 40.7% of cases, there was evidence of IH on brain MRI, but no CSF leak or extradural CSF collection. A further 15.1% had inconclusive findings and 10.5% had no evidence of IH or CSF leak on radiological imaging.

**Table 3:** Diagnostic procedure for SIH

Examination/category	MRI brain/spine	CT myelography	MR myelography	Optic nerve sheath diameter	CT cisternography	CSF scintigraphy	CSF pressure
	*n* (%)	*n* (%)	*n* (%)	*n* (%)	*n* (%)	*n* (%)	*n* (%)
**Performed**							
Yes	86 (100)	51 (59.3)	22 (25.6)	12 (14.0)	22 (25.6)	4 (4.7)	39 (45.3)
No	0	35 (40.7)	64 (74.4)	74 (86.0)	64 (74.4)	82 (95.3)	47 (54.7)
**Interval since onset**							
< 1 month	34 (39.5)	3 (5.9)	–	–	–	–	2 (5.1)
1–3 months	28 (32.6)	5 (9.8)	3 (13.6)	6 (50.0)	3 (13.6)	1 (25.0)	10 (25.6)
3–6 months	8 (9.3)	15 (29.4)	5 (22.7)	1 (8.3)	5 (22.7)	–	9 (23.1)
> 6 months	16 (18.6)	28 (54.9)	14 (63.6)	5 (41.7)	14 (63.6)	3 (75.0)	18 (46.2)
**Number of examinations**							
1	–	1 (2.0)	1 (4.5)	1 (8.3)	3 (13.6)	1 (25.0)	–
2	9 (10.5)	29 (56.9)	5 (22.7)	7 (58.3)	17 (77.3)	3 (75.0)	22 (56.4)
3	33 (38.4)	19 (37.3)	11 (50.0)	2 (16.7)	2 (9.1)	–	11 (28.2)
4	44 (51.2)	2 (3.9)	5 (22.7)	2 (16.7)	–	–	6 (15.4)

Table 4 analyzes the different treatments for SIH patients. Participants without radiological evidence were more likely to use analgesics (92.6%) than those with radiological evidence (84.7%). Theophylline use was less common in both groups, but slightly higher in those without radiological evidence (29.6% vs. 18.6%). Gabapentin was used by 42.4% of those with radiological evidence and 59.3% of those without. In terms of duration of use, participants without radiological evidence tended to use drugs for longer periods, especially over 12 months (56.0% vs. 36.0% for analgesics). In terms of short-term effectiveness, many people found that treatment, such as analgesics, theophylline and gabapentin had little or no effect.

**Table 4:** Evaluation and comparison of the effects of different medications in relieving symptoms in patients with and without radiological evidence of SIH

	With radiological evidence *n* = 59			Without radiological evidence *n* = 27		
Category	Analgesics *n* (%)	Theophylline *n* (%)	Gabapentin *n* (%)	Analgesics *n* (%)	Theophylline *n* (%)	Gabapentin *n* (%)
**Performed**						
Yes	50 (84.7)	11 (18.6)	25 (42.4)	25 (92.6)	8 (29.6)	16 (59.3)
No	9 (15.3)	48 (81.4)	34 (57.6)	2 (7.4)	19 (70.4)	11 (40.7)
**Duration of use**						
< 1 week	4 (8.0)	4 (36.4)	1 (4.0)	1 (4.0)	1 (12.5)	1 (6.3)
2–4 weeks	2 (4.0)	2 (18.2)	6 (24.0)	4 (16.0)	2 (25.0)	4 (25.0)
1–3 months	13 (26.0)	3 (27.3)	7 (28.0)	6 (24.0)	4 (50.0)	6 (37.5)
3–6 months	4 (8.0)	2 (18.2)	3 (12.0)	2 (8.0)	1 (12.5)	2 (12.5)
6–12 months	9 (18.0)	0	6 (24.0)	3 (12.0)	0	3 (18.8)
Over 12 months	18 (36.0)	0	2 (8.0)	14 (56.0)	0	0
**Short-term efficacy**						
Not effective	25 (50.0)	7 (63.6)	14 (56.0)	5 (20.0)	2 (25.0)	10 (62.5)
Slightly	21 (42.0)	2 (18.2)	5 (20.0)	9 (36.0)	5 (62.5)	3 (18.8)
Moderately	2 (4.0)	2 (18.2)	1 (4.0)	6 (24.0)	1 (12.5)	2 (12.5)
Effective	2 (4.0)	0	5 (20.0)	5 (20.0)	0	1 (6.3)
Completely effective	0	0	0	0	0	0
**Long-term efficacy**						
Not effective	37 (74.0)	8 (72.7)	19 (76.0)	13 (52.0)	5 (62.5)	13 (81.3)
Slightly	11 (22.0)	3 (27.3)	1 (4.0)	4 (16.0)	3 (37.5)	2 (12.5)
Moderately	1 (2.0)	0	2 (8.0)	7 (28.0)	0	1 (6.3)
Effective	1 (2.0)	0	3 (12.0)	1 (4.0)	0	0
Completely effective	0	0	0	0	0	0
**Side effects**						
No side effects	34 (68.0)	6 (54.5)	5 (20.0)	11 (44.0)	5 (62.5)	4 (25.0)
Mild	14 (28.0)	3 (27.3)	3 (12.0)	10 (40.0)	3 (37.5)	2 (12.5)
Moderate	2 (4.0)	1 (9.1)	9 (36.0)	4 (16.0)	1 (12.5)	2 (12.5)
Severe	0	1 (9.1)	1 (4.0)	2 (8.0)	0	2 (12.5)
Very severe	0	0	0	0	0	0

In Table 5 all 86 participants reported having received an EBP. The group with radiological evidence received 1–2 EBP in 40% of cases and almost a further 40% received 3–4 treatments. In contrast, the group without radiological evidence received a comparable number of 3–4 treatments; however, almost half of the participants received 5–7 EBP. The short-term effectiveness of EBP was reported as mild by almost 40% and moderate by one third of all participants. In the long term, almost 40% reported mild effectiveness and almost 18% reported moderate effectiveness.

**Table 5:** A comparative analysis of the efficacy of various invasive therapeutic procedures in relieving symptoms in patients with and without radiological evidence of SIH

	With radiological evidence *n* = 59			Without radiological evidence *n* = 27		
**Category**	Epidural blood patch *n* (%)	Occipital nerve block *n* (%)	Surgery *n* (%)	Epidural blood patch *n* (%)	Occipital nerve block *n* (%)	Surgery *n* (%)
**Performed**						
Yes	59 (100)	11 (18.6)	29 (49.1)	27 (100)	12 (44.4)	0
No	0	48 (81.4)	30 (51.9)	0	15 (55.6)	27 (100)
**Number of treatments**						
1–2	24 (40.6)	8 (72.7)	29 (100)	5 (18.5)	3 (25.0)	0
3–4	22 (37.3)	1 (9.1)	0	9 (33.3)	2 (16.7)	0
5–6	9 (15.3)	0	0	7 (25.9)	2 (16.7)	0
6–7	4 (6.8)	2 (18.2)	0	6 (22.2)	5 (41.7)	0
**Short-term efficacy**						
Not effective	6 (10.2)	5 (45.5)	5 (17.2)	0	3 (25.0)	0
Slightly	22 (37.3)	4 (36.4)	4 (13.8)	13 (48.1)	8 (66.7)	0
Moderate	22 (37.3)	2 (18.2)	12 (41.4)	7 (25.9)	1 (8.3)	0
Effective	4 (6.8)	0	6 (20.7)	5 (18.5)	0	0
Completely effective	5 (8.5)	0	3 (10.3)	1 (3.7)	0	0
**Long-term efficacy**						
Not effective	17 (28.8)	5 (45.5)	5 (17.2)	11 (40.7)	3 (25.0)	0
Slightly	24 (40.7)	4 (36.4)	3 (10.3)	10 (37.0)	9 (75.0)	0
Moderate	13 (22.0)	2 (18.2)	5 (17.2)	3 (11.1)	0	0
Effective	2 (3.4)	0	12 (41.4)	2 (7.4)	0	0
Completely effective	3 (5.1)	0	4 (13.8)	0	0	0
**Side effects**						
No side effects	10 (16.9)	6 (54.5)	9 (31.1)	7 (25.9)	6 (50.0)	0
Mild	16 (27.1)	4 (36.4)	6 (20.7)	9 (33.3)	4 (33.3)	0
Moderate	22 (37.3)	1 (9.1)	11 (37.9)	8 (29.6)	2 (16.7)	0
Severe	6 (10.2)	0	2 (6.9)	2 (7.4)	0	0
Very severe	5 (8.5)	0	1 (3.4)	0	0	0

Occipital nerve blocks were administered to almost one quarter of all participants. In both groups, nearly 80% of participants reported that short-term and long-term therapy yielded no or only minor benefits. Half of the participants reported no side effects, while the other half reported mild to moderate side effects from the occipital nerve block.

Of the 29 people who opted for targeted surgery after confirmation of a CSF leak, 3 participants reported undergoing intradural exploratory surgery without a precise location of the leak, but with evidence of extradural CSF collection on MRI. In the short term, surgery was found to be moderately effective in about half of the participants. In the long term, it was effective in 34.5% of cases and completely effective in 13.8%. In addition, almost 40% of participants reported moderate side effects, while just over 10% reported severe to very severe side effects from surgery. Of these, 8 people reported severe back pain, 5 people experienced intraspinal bleeding and 1 person was diagnosed with chronic adhesive arachnoiditis.

Table 6 explores changes in relationships, emotional distress, disability, and mental states due to SIH. Over 20% of the participants struggled with significant relationship difficulties, with some cases (over 10%) even leading to separation or divorce, reflecting the substantial emotional impact of the condition. Over half of participants reported emotional distress due to SIH, highlighting its significant emotional impact. Participants without radiological evidence reported higher levels of disability, with 63% experiencing severe disability compared to 30.5% in the group with radiological evidence. Only 14.8% of those without radiological evidence reported mild disability, whereas almost half of those with radiological evidence reported mild disability. Regarding the DASS21 scores for depression, the group without radiological evidence had a higher median score of 24 (interquartile range, IQR 7–28) compared to 15 (IQR 7–28) in the group with radiological evidence. Severe depression score was reported by 40.7% of those without radiological evidence, compared to 16.9% in the group with radiological evidence. For anxiety, the median score was similar between the groups, with 15 (IQR 7–27) for those with radiological evidence and 12 (IQR 7–27) for those without. In terms of stress, the median scores were identical, with 15 (IQR 7–28) for those with radiological evidence and 15 (IQR 8–28) for those without. Mild stress was more prevalent in the group without radiological evidence (70.4% vs. 47.5%) (Table 6).

**Table 6:** Frequency and percentage of changes in relationships, emotional distress, disability and mental state

Variable	All participants (*n* = 86)	With radiological evidence (*n* = 59)	Without radiological evidence (*n* = 27)
**Changes or difficulties in relationship, ***n* (%)			
No noticeable impact	12 (14.0)	8 (13.6)	4 (14.8)
Yes, worked through problems	22 (25.6)	15 (25.4)	7 (25.9)
Yes, significant difficulties	20 (23.3)	16 (27.1)	4 (14.8)
Yes, led to separation/divorce	10 (11.6)	6 (10.2)	4 (14.8)
Prefer not to answer	22 (25.6)	14 (23.7)	8 (29.6)
**Emotional distress due to SIH, *n* (%)**			
Yes, I feel a lot of emotional distress	45 (52.3)	29 (49.2)	16 (59.3)
Emotionally distressed, but not severely	26 (30.2)	19 (32.2)	7 (25.9)
Not sure if distressed	15 (17.4)	11 (18.6)	4 (14.8)
No, I have not noticed any negative impact	0	0	0
**Disability index, *n* (%)**			
Mild disability	32 (37.2)	28 (47.5)	4 (14.8)
Moderate disability	17 (19.8)	11 (18.6)	6 (22.2)
Severe disability	35 (40.7)	18 (30.5)	17 (63.0)
Complete disability	2 (2.3)	2 (3.4)	0
**DASS21 score depression; median (IQR), *n* (%)**	17 (7–28)	15 (7–28)	24 (7–28)
Normal	19 (22.1)	15 (25.4)	4 (14.8)
Mild	13 (15.1)	10 (16.9)	3 (11.1)
Moderate	22 (25.6)	17 (28.8)	5 (18.5)
Severe	21 (24.4)	10 (16.9)	11 (40.7)
Extremely severe	11 (12.8)	7 (11.9)	4 (14.8)
**DASS21 score anxiety; median (IQR), *n* (%)**	12 (7–27)	15 (7–27)	12 (7–27)
Normal	3 (3.5)	2 (3.4)	1 (3.7)
Mild	18 (20.9)	11 (18.6)	7 (25.9)
Moderate	34 (39.5)	24 (40.7)	10 (37.0)
Severe	16 (18.6)	12 (20.3)	4 (14.8)
Extremely severe	15 (17.4)	10 (16.9)	5 (18.5)
**DASS21 score stress; median (IQR), *n* (%)**	15 (7–28)	15 (7–28)	15 (8–28)
Normal	9 (10.5)	8 (13.6)	1 (3.7)
Mild	47 (54.7)	28 (47.5)	19 (70.4)
Moderate	22 (25.6)	18 (30.5)	4 (14.8)
Severe	8 (9.3)	5 (8.5)	3 (11.1)
Extremely severe	0	0	0

**Fig. 1:**
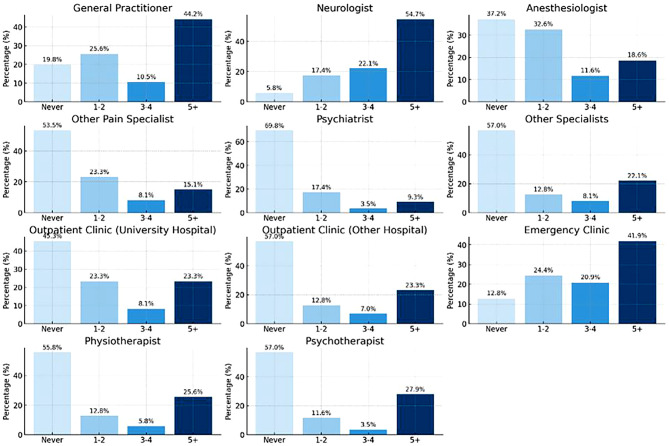
Consultation of physicians. Note: This figure shows the frequency of consultations with different healthcare providers, including general practitioners, neurologists, anaesthetists and others. The bars show the percentage of participants and the number of consultations: “never”, “1-2”, “3-4” or “5+”

**Fig. 2:**
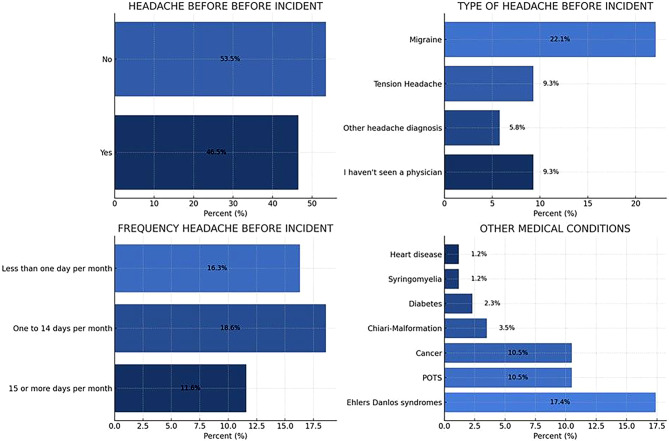
Headaches preceding the onset of SIH and other medical conditions. Note: This figure shows the history of headache and other medical conditions before the onset of spontaneous intracranial hypotension (SIH). It includes the prevalence, type and frequency of headaches and other conditions such as postural orthostatic tachycardia syndrome (POTS) and Ehlers-Danlos syndrome

Patients frequently visit general practitioners, with almost half reporting more than 5 visits, around a quarter 1–2 visits, 10.5% 3–4 visits and almost a fifth never. Neurologists see more than half of their patients more than 5 times, 22.1% visit 3–4 times, 17.4% visit 1–2 times and only 5.8% never visit. For anesthetists, 18.6% of patients reported more than 5 visits, 11.6% visited 3–4 times, about one third (32.6%) visited 1–2 times and more than one third (37.2%) never visited. Emergency departments were visited more than 5 times by 41.9% of patients, almost one quarter (24.4%) never visited, 20.9% visited 1–2 times and 12.8% visited 3–4 times. Detailed visit distributions are shown in Fig. 2.

In addition, as shown in Fig. 2, almost half of the participants experienced headaches before the onset of SIH, with migraine being the most common type reported. Headaches occurred up to 14 days per month in a significant number of individuals. In addition, Ehlers-Danlos syndrome and postural tachycardia syndrome were the most commonly reported comorbid conditions, each affecting over 10% of the cohort.

## Discussion

The present study contributes to understanding the severe impact of SIH, a condition that, despite its rarity, has profound consequences for affected individuals. Studies [19–21] indicated that diagnosing SIH is particularly challenging in patients with a history of chronic headaches. The process often extends over a longer period, especially when the characteristic orthostatic headache is not a prominent feature. These difficulties in diagnosing SIH may also be reflected in our cohort, where approximately one third of participants had chronic headaches prior to the onset of SIH and 65% of participants did not receive a diagnosis of SIH from a physician for more than 3 months. These delays underscore the necessity for enhanced diagnostic awareness and accelerated identification procedures to mitigate the multifaceted socioeconomic and health consequences in individuals diagnosed with SIH. This dichotomized analysis revealed two distinct groups: patients with radiological evidence of SIH and those without. This distinction highlights the diagnostic dilemma frequently encountered in clinical practice, where the absence of clear radiological evidence complicates SIH management. This challenge is common in SIH research and clinical settings, as studies have shown the complexity and variability of SIH presentations [21].

The long-term effects of SIH are underscored by the substantial proportion of participants who, since the onset of SIH, are either unable to work or have been forced into retirement, over one third of the cohort. Additionally, the fact that one third of respondents have been on sick leave for more than 1 year highlights the condition’s significant socioeconomic impact. This observation mirrors findings from previous studies that have pointed to the substantial effects of SIH on work capacity, as noted by Cheema et al. [12]. Moreover, the data show that over half of the respondents have been living with SIH for more than 3 years, underlining the chronic nature of this condition and echoing observations by Kranz et al. [22], which indicate the potential for persistent and intractable symptomatology associated with SIH. The deterioration of the condition reported by one third of the participants since the onset of the illness emphasizes the need for effective long-term treatment.

Our data reveal that most patients (40.7%) classified their headaches as severe, while 14% described them as extremely severe. This severe pain, combined with other debilitating symptoms, such as nausea, dizziness, tinnitus and balance problems, is also reflected in the psychological profile of those affected. Moreover, the fact that more than half have coped with SIH for over 3 years underscores the disease’s unrelenting nature. Analysis of the DASS-21 data, which consists of 3 self-report scales designed to measure the emotional states of depression, anxiety and stress, shows that 77.9% of participants reported elevated levels of depression, 96.5% reported elevated levels of anxiety and 89.5% reported elevated levels of stress according to the DASS-21 subscales. Further breakdown shows that approximately 37.2% of participants scored in the severe or extremely severe range for depression, approximately 36% for anxiety and 9.3% for stress on the DASS-21 severity scales. It is important to note that these results reflect self-reported emotional states rather than clinical diagnoses. For comparison, Cheema et al. indicated in their study that SIH patients have an average EuroQol-5 Dimension Visual Analog Scale score (EQ-5D VAS) of 36.4, highlighting substantial impairment. This score falls below those for other severe conditions, such as multiple sclerosis (59.7) and advanced Parkinson’s disease (52.0), underscoring the disproportionately negative impact on QoL for those with SIH. In contrast, a recently published study [23] reported significant improvements in health-related quality of life (HRQoL), measured by the EQ-5D-5L, and mental health, assessed using the DASS-21, in SIH patients following surgical closure of a spinal CSF leak. The EQ-Index increased from 0.683 preoperatively to 0.907, 6 months postoperatively, and the EQ-VAS improved from 40 to 72 over the same period. The study found that a relevant symptom burden due to depression, anxiety, and stress was highly prevalent among the patients and significantly improved after treatment. This suggests that patients’ psychological distress is largely a reaction to their physical illness. In contrast to the findings of Volz et al. [23], where the median duration of symptoms among participants was 5 months, our data show a longer duration of illness. This prolonged exposure to stressors associated with the illness could explain the higher DASS-21 scores for depression, anxiety, and stress observed in our study. Extended periods of illness are often linked with increased psychological stress, as they can lead to sustained activation of stress response systems [24]. This condition likely contributes to the differences in the psychometric profiles between the two study populations. Additionally, Volz et al. [24] reported better outcomes, which may be because only SIH patients with a confirmed leak were included in their study, whereas our cohort included patients with and without a confirmed leak. Furthermore, the higher number of patients who underwent surgery in their study compared to our study, where only about one third of the participants underwent surgery, could also explain the differences in outcomes.

However, another study revealed that nearly half of their cohort exhibited moderate depression and more than one in four showed moderate anxiety [25]. In this cohort, the frequency of suicidal thoughts and behavior was notably higher than in migraine patients and similar to that observed in cluster headache patients. In detail, over half of the patients expressed at least a wish to be dead and nearly one quarter acknowledged previous suicidal behavior. During patient interviews, many directly attributed their suicidality to their headache pain and the fear of never improving, although such disclosures were not formally recorded. Reflecting the depth of this impact, our research also found that the majority of the cohort felt deeply distressed by their ongoing battle with SIH. Moreover, our study indicated that SIH is not only a physical and mental ordeal but also one that deeply affects personal relationships. Nearly one quarter of participants reported significant relational strains, as shown in Table 6, with 11.6% experiencing break-ups or divorces. This highlights the profound stress that chronic diseases can impose on social and family life. These insights are in line with research that underscores the impact of long-standing pain and chronic conditions on relationships [13, 26]. This distressing finding points to a critical need for mental health support as an integral component of SIH care.

Our study’s findings necessitate a layered diagnostic approach to SIH, as encapsulated in Table 4, which underscores the complexity of clinical decision-making in this context. The data indicate that nearly 40% of participants received an MRI scan within 1 month of presenting SIH symptoms, with just over one third undergoing brain or spine MRI within 1–3 months. Approximately one quarter of the individuals received an MRI after 3 months. For more invasive procedures such as CT myelography, a higher proportion of tests were performed at 3 months. While a dynamic examination, such as dynamic CT myelography is essential for accurate localization of the CSF leak, especially when MRI alone is not sufficient to locate the exact site of the leak, our study shows that these invasive procedures are used less frequently. This may indicate a cautious approach due to the invasiveness of the procedures, or it may reflect complex cases where initial diagnostic attempts were inconclusive or where symptoms changed over time; however, it is also important to note that the results of dynamic myelograms are highly dependent on the experience of the performing physician [27]. Given the categorical nature of our data, it is difficult to directly compare median waiting times with those reported by Cheema et al. [12]. In their study, the median waiting time for MRI was 4 weeks (IQR: 11 weeks, range: 0–56 weeks), which is comparable to our cohort. A CT myelography was performed in 52.9% of their patients, a similar percentage to ours, but the median waiting time for CT myelography in Cheema’s cohort was 20 weeks (IQR: 36 weeks). The diagnostic results showed that CSF leakage or extradural CSF collection was identified in almost one third of participants, while 40.7% had evidence of IH on brain MRI without evidence of CSF leakage or extradural CSF collection. A further 15.1% had inconclusive findings, while 10.5% had no evidence of IH or CSF leakage on radiological imaging. This aligns with findings from a systematic review [8], which notes that 19% of SIH patients have normal brain and spine MRI results, presenting a considerable diagnostic challenge. The variation in the sensitivity of spinal investigations for CSF leaks, with detection rates of extradural CSF between 48% and 76%, suggests that a substantial number of SIH cases might initially go undetected. Moreover, the same review indicated that 24% of cases involve multiple leaks, underscoring the necessity for a thorough and comprehensive approach in diagnostic imaging, further compounding the complexity of diagnosis.

In assessing the impact of various medications on SIH, the data show that analgesics and caffeine are the most commonly used treatments, with high administration rates (87.2% and 89.5%, respectively); however, their effectiveness appears to be limited, with a significant proportion of patients reporting minimal long-term relief. The high rate of analgesic use in our study suggests possible overuse of medication, which is a common problem in the treatment of chronic pain, as other studies have shown [28]. Patients often continue to take medication in the hope of relief, despite limited benefit. Such overuse may lead to further complications and highlights the need for more effective and targeted treatment strategies for SIH. The use of EBP in managing SIH reveals a complex picture of treatment efficacy. In our study, EBPs were frequently utilized, though the outcomes varied. Initial relief was achieved in a minority of patients, with complete and moderate improvement noted in 7% and just over one third of patients, respectively; however, the majority experienced little or no substantial benefit from the intervention, a trend that remained stable in the long term. Studies show varying treatment efficacies: 1 study [8] reported that 55.9% of patients experienced complete resolution of their symptoms after a single EBP, while another study [29] indicated that about 70% of patients still showed evidence of a persistent leak after EBP. Factors such as the volume of blood injected, the timing of the procedure, and individual patient characteristics can influence the effectiveness of EBPs. Therefore, a patient who continues to show symptoms after an EBP should be referred for adequate diagnostics and treatment at a specialized center to pursue targeted treatment. A prolonged duration of over 12 weeks [13] can increase the likelihood of chronicity and complications. Our data also indicate differences in the short-term and long-term effectiveness of various treatments. While some patients experienced improvements, it remains unclear which specific treatment in the sequence was most important. Despite multiple treatments, a significant number of patients did not show notable long-term improvement in their condition.

Surgery emerged as a significant intervention in the management of SIH, indicated in over one third of patients in our study. This rate of surgical intervention may reflect the complex nature of SIH in these patients or a limited response to evidence-based practices. Although there was an overall reduction in disability and headache intensity after surgery, some patients reported persistent symptoms. Consistent with the findings of Volz et al. [11], around one quarter of the patients in their cohort continued to suffer from considerable symptoms after 1 year. This persistence suggests that although surgery provides significant symptom relief for many individuals, it is not a panacea. The reasons for this are complex and may include factors such as the development of rebound intracranial hypertension or coexisting headache disorders. In the present study, 3 participants underwent intradural exploratory surgery without the precise localization of the CSF leak. This type of surgery presents significant challenges, increasing the procedure’s duration and complexity, as well as the risk of complications such as infection and bleeding. Consequently, patients frequently experience more severe postoperative pain and longer recovery times. Furthermore, there is an increased probability of the leak being undetected or inadequately repaired, necessitating additional surgical intervention. The necessity for additional intraoperative diagnostic procedures may further complicate and prolong the surgical procedure [30].

Analysis of specialist consultations for SIH patients shows that they frequently visit general practitioners and neurologists, indicating the need for ongoing management of persistent symptoms. The high frequency of visits to emergency departments, as shown in Fig. 2, also indicates that these patients significantly suffer from the physical effects of their condition. Despite the obvious psychological distress, the use of psychiatric services by SIH patients remains relatively low. This may indicate that psychological symptoms are often perceived as a reactive state to unresolved physical symptoms. Research, such as the recently published study by Volz et al. [23], suggests that psychological symptoms may subside if the physical complaints are effectively treated or alleviated. This underscores the importance of a holistic approach to treatment that comprehensively addresses both the physical and psychological aspects of SIH.

### Limitations

Our study, designed as a cross-sectional online survey, faced certain limitations. A significant concern is the potential influence of recall and self-report biases, which are inherent challenges when participants are tasked with retrospectively reporting medical histories, including treatments and diagnoses. This is particularly relevant as the severity and impact of SIH can change over time. Furthermore, selection bias presents a significant challenge in our study due to the recruitment of participants through online self-help groups. This method tends to attract individuals who may be actively seeking support or experiencing more severe symptoms. As a result, the findings could be skewed towards a higher severity of the condition, affecting the generalizability of our results across the broader SIH population. Compounding this issue is the absence of a control group entirely cured of SIH, which is intrinsically linked to the selection bias concern. The lack of such a control group limits the capacity to accurately assess the effectiveness of certain treatments and to validate some of the study conclusions. A control group, consisting of individuals cured of SIH, would have offered a crucial benchmark for evaluating the success of treatments and the progression of the disease, thereby providing a more comprehensive understanding of SIH’s dynamics and treatment responses. Although the inclusion criteria of the study were relatively strict, requiring participants to have undergone at least one EBP treatment and to have had at least one MRI scan of both the spine and the brain, there remains the possibility that some individuals without SIH have been included. This may affect the accuracy of the data; however, as described previously, studies [9, 10] have shown that different spinal imaging techniques were only successful in identifying evidence of extradural CSF leaks in a limited range of 48–67% of individuals, which is comparable to our study.

## Conclusion

This comprehensive study shows that SIH is not only a serious mental health burden, but also has a significant socioeconomic impact. Many patients face extended periods of illness and a considerable reduction in their work capacity, highlighting the need for a holistic treatment approach that includes mental health support alongside physical healthcare. Simultaneously, the study sheds light on the varying effectiveness of treatments like EBP and surgical interventions. While these treatments show promise in symptom relief, the long-term outcomes are inconsistent, with many patients continuing to experience symptoms after treatment. This inconsistency underscores an urgent need for the development of more targeted and effective therapeutic strategies that can address the complex and layered nature of SIH. Therefore, the study emphasizes the necessity for continuous research and innovation in the diagnostic and therapeutic aspects of SIH to improve the quality of life for patients suffering from this challenging condition.

## Appendix

**Table 7:** Checklist for Reporting Results of Internet E‑Surveys (CHERRIES) for SIH survey

Item category	Checklist item	Explanation
*Design*	Survey design	Cross-sectional online survey focused on the experiences of individuals with SIH. Conducted globally, the survey consisted of 5 sections: Sect. 1—Demographics and Characteristics of SIH, Sect. 2—Treatment, Sect. 3—Diagnostic, Sect. 4—The Depression Anxiety Stress Scale-21, and Sect. 5—Hospital Headache Disability Inventory. The survey comprised 106 questions across 20 pages, Survey questions are listed in Appendix B
*IRB approval and informed consent process*	IRB approval	Ehical approval obtained from the Medical University of Vienna, approval number 1180/2023
	Informed consent	An online consent process where participants clicked “Agree” to signify understanding and meeting inclusion criteria. Here is the text for consent: “Dear Sir/Madam, We cordially invite you to participate in our online survey. Participation in this anonymous study is voluntary, and you may withdraw from it at any time without providing a reason. Refusing to participate or withdrawing early from the study will not have any adverse consequences for you. The purpose of the survey is to better understand the challenges and needs of individuals with SIH. The development of this type of headache can lead to significant impairments, including lost work days, unemployment, inability to work, and reduced quality of life (QOL). As a participant, you will have the opportunity to share your own perspectives and experiences with SIH and other related symptoms. This study is conducted on behalf of the Medical University of Vienna, Center for Public Health at the Institute for Social and Preventive Medicine. Your participation in the study is voluntary, and your data will be treated as anonymous and confidential. No personal information, such as IP address, name, or email address, will be collected or stored. The study is expected to take approximately 30 min to complete. By clicking on “Next” at the bottom right, you automatically agree to participate in this study. We aim to have at least 100 participants take part in this study. If you have any further questions or concerns, please do not hesitate to contact the project lead, Ali Kapan, at the Medical University of Vienna, Center for Public Health, Institute for Social and Preventive Medicine.”
*Data protection*	Data protection	Data was collected using the SoSci Survey platform, known for its strong emphasis on data security and privacy. The platform ensures that all data transfer is encrypted using SSL (HTTPS) protocols. Additionally, data storage and processing conform to the stringent regulations of the GDPR, as the servers are located in Germany. The entire data analysis process was conducted on encrypted devices to further safeguard the integrity and confidentiality of the data collected. No personally identifiable information, such as names or IP addresses, was collected, ensuring participant anonymity and privacy
*Development and pretesting*	Development and testing	The development of the survey was a collaborative effort, involving clinical researchers, SIH patients, and experts in the field of headaches. Notably, one of the authors, a patient with personal experience of chronic Postdural puncture Headache, contributed significantly to the survey design, ensuring a patient-centered approach and relevance. Another author, leading the headache department at the AKH Vienna, brought extensive clinical expertise to the project. This combination of personal experience and professional expertise was instrumental in formulating questions that accurately capture the complexities of SIH. The survey was pilot tested to assess its technical functionality and the clarity of its questions, ensuring it effectively addressed the key research objectives
*Recruitment process and sample description*	Open vs. closed survey	This was a closed survey, targeting a specific demographic—adults diagnosed or suspected with SIH
	Contact mode	The survey was promoted through social media platforms of specific organizations related to SIH reaching an audience of over 17,000 followers. This approach utilized the unique communities and networks within each group, ensuring a more focused and effective recruitment. The survey link, https://www.soscisurvey.de/csfleak/, was shared on these platforms, and group administrators played a key role in monitoring and facilitating the survey’s distribution, thereby enhancing its visibility and participation rate among relevant individuals
	Advertising the survey	Prior to announcing the survey on social media, the research team sought permission from administrators of relevant Facebook groups. Upon receiving approval, a detailed survey advertisement was posted in each group. This advertisement included comprehensive information about the study, highlighting its purpose and voluntary nature. The post also contained a direct link to the survey and information about informed consent. This process ensured ethical conduct and respect for the group’s norms, while effectively reaching the target audience for the survey
*Survey administration*	Web/E-mail	The survey was hosted online, with a specific URL (https://www.soscisurvey.de/csfleak/). The interface was customized for user-friendliness and clarity
	Context	No specific website was used; the survey was directly accessible via the provided URL
	Mandatory/voluntary	Participation was voluntary, with a detailed explanation and consent process at the beginning
	Incentives	No incentives were offered; participation was entirely voluntary and aimed at academic research
	Time/date	The survey was active from April 19 to June 19, 2023. To ensure continuous engagement, weekly reminders about the survey were periodically posted on these platforms
	Randomization of items	Questions were presented in a fixed order for each participant
	Adaptive questioning	Adaptive questioning principles were used to number and handle the complexity of the questions
	Number of items	The survey consisted of 5 sections: Sect. 1—Demographics and Characteristics of SIH, Sect. 2—Treatment, Sect. 3—Diagnostic, Sect. 4—The Depression Anxiety Stress Scale-21, and Sect. 5—Hospital Headache Disability Inventory. The survey comprised 106 questions across 20 pages
	Number of screens	Survey distributed over 20 pages
	Completeness check	The survey incorporated both mandatory and optional questions. Clinical characteristic questions required a response, ensuring critical data were thoroughly collected. In contrast, standardized questionnaires allowed skipping questions, offering flexibility to participants. Additionally, all questions featured “not applicable” or “don’t know” options, accommodating varied respondent experiences and maintaining data integrity
	Review step	Respondents were able to move between pages to change responses. However, once they submitted the survey the responses were final
*Response rates*	Unique site visitor	Total of 1448 clicks, including unintentional clicks
	View rate	Not calculated
	Participation rate	A total of 347 individuals consented to participate in the survey
	Completion rate	86 out of 347 participants completed the survey
*Preventing multiple entries*	Cookies and IP check	No use of cookies; IP addresses were not collected for duplicate entry checks
	Log file analysis	Not applicable
	Registration	Each participant accessed the survey via unique online link
*Analysis*	Handling incomplete Questionnaires	Only fully completed surveys were analyzed
	Questionnaires with atypical time stamp	Not applicable
	Statistical correction	No specific statistical correction methods were employed in the analysis of our survey data. Given the descriptive nature of our study, our focus was on presenting straightforward, unweighted frequencies and percentages to accurately reflect the responses received. Our approach was to provide a clear and direct representation of the data as collected, without applying additional statistical adjustments or weighting. This approach aligns with our objective to offer a descriptive overview of the participants’ responses, rather than inferential or predictive analyses. As a result, our data analysis primarily involved basic descriptive statistics, ensuring a transparent and direct presentation of the findings

## References

[1] Schievink WI. Spontaneous Intracranial Hypotension. N Engl J Med. 2021;385(23):2173–8. 34874632 10.1056/NEJMra2101561

[2] Chen S, Hagiwara M, Roehm PC. Spontaneous intracranial hypotension presenting with severe sensorineural hearing loss and headache. Otol Neurotol Off Publ Am Otol Soc Am Neurotol Soc [and] Eur Acad Otol Neurotol. 2012;33(8):e65. 10.1097/MAO.0b013e318254ed98 PMC3600858 22722142

[3] Kranz PG, Gray L, Malinzak MD, Amrhein TJ. Spontaneous intracranial hypotension: pathogenesis, diagnosis, and treatment. Neuroimaging Clin N Am. 2019;29(4):581–94. 31677732 10.1016/j.nic.2019.07.006

[4] Dobrocky T, Nicholson P, Häni L, Mordasini P, Krings T, Brinjikji W, Cutsforth-Gregory JK, Schär R, Schankin C, Gralla J. Spontaneous intracranial hypotension: searching for the CSF leak. Lancet Neurol. 2022;. 10.1016/S1474-4422(21)00423-3 35227413

[5] Headache Classification Committee of the International Headache Society (IHS). The International Classification of Headache Disorders. Cephalalgia. 2018;38(1):1–211. 3rd edition. 10.1177/0333102417738202 29368949

[6] Kranz PG, Tanpitukpongse TP, Choudhury KR, Amrhein TJ, Gray L. How common is normal cerebrospinal fluid pressure in spontaneous intracranial hypotension? Cephalalgia. 2016;36(13):1209–17. 26682575 10.1177/0333102415623071

[7] Beck J, Gralla J, Fung C, Ulrich CT, Schucht P, Fichtner J, Andereggen L, Gosau M, Hattingen E, Gutbrod K. Spinal cerebrospinal fluid leak as the cause of chronic subdural hematomas in nongeriatric patients. JNS. 2014;121(6):1380–7. 10.3171/2014.6.JNS14550 25036203

[8] D’Antona L, Merchan MAJ, Vassiliou A, Watkins LD, Davagnanam I, Toma AK, Matharu MS. Clinical presentation, investigation findings, and treatment outcomes of spontaneous intracranial hypotension syndrome: a systematic review and meta-analysis. JAMA Neurol. 2021;78(3):329–37. 33393980 10.1001/jamaneurol.2020.4799 PMC7783594

[9] Beck J, Raabe A, Schievink WI, Fung C, Gralla J, Piechowiak E, Seidel K, Ulrich CT. Posterior approach and spinal cord release for 360 repair of dural defects in spontaneous intracranial hypotension. Neurosurgery. 2019;84(6):E345–E51. 30053151 10.1093/neuros/nyy312

[10] Wang TY, Karikari IO, Amrhein TJ, Gray L, Kranz PG. Clinical outcomes following surgical ligation of cerebrospinal fluid-venous fistula in patients with spontaneous intracranial hypotension: a prospective case series. Oper Neurosurg. 2020;18(3):239–45. 10.1093/ons/opz134 31134267

[11] Volz F, Fung C, Wolf K, Lützen N, Urbach H, Kraus LM, Omer M, Beck J, El Rahal A. Recovery and long-term outcome after neurosurgical closure of spinal CSF leaks in patients with spontaneous intracranial hypotension. Cephalalgia. 2023;43(8):3331024231196808. 10.1177/03331024231195830 37652456

[12] Cheema S, Joy C, Pople J, Snape-Burns J, Trevarthen T, Matharu M. Patient experience of diagnosis and management of spontaneous intracranial hypotension: a cross-sectional online survey. BMJ Open. 2022;12(1):e57438. 35058269 10.1136/bmjopen-2021-057438 PMC8783814

[13] Jesse CM, Häni L, Fung C, Ulrich CT, Schär RT, Dobrocky T, Piechowiak EI, Goldberg J, Schankin C, Sintonen H, et al. The impact of spontaneous intracranial hypotension on social life and health-related quality of life. J Neurol. 2022;269(10):5466–73. 35701531 10.1007/s00415-022-11207-7 PMC9467959

[14] Eysenbach G. Improving the quality of Web surveys: the Checklist for Reporting Results of Internet E‑Surveys (CHERRIES). J Med Internet Res. 2004;6(3):e34. 15471760 10.2196/jmir.6.3.e34 PMC1550605

[15] Callen AL, Friedman DI, Parikh S, Rau JC, Schievink WI, Cutsforth-Gregory JK, Amrhein TJ, Haight E, Cowan RP, Barad MJ. Diagnosis and Treatment of Spontaneous Intracranial Hypotension: Role of Epidural Blood Patching. Neurol Clin Pract. 2024;14(3):e200290. 38699599 10.1212/CPJ.0000000000200290 PMC11065326

[16] Lovibond SH. Manual for the depression anxiety stress scales. Sydney: psychology foundation; 1995.

[17] Ng F, Trauer T, Dodd S, Callaly T, Campbell S, Berk M. The validity of the 21-item version of the Depression Anxiety Stress Scales as a routine clinical outcome measure. Acta Neuropsychiatr. 2007;19(5):304–10. 26952943 10.1111/j.1601-5215.2007.00217.x

[18] Jacobson GP, Ramadan NM, Aggarwal SK, Newman CW. The Henry Ford Hospital Headache Disability Inventory (HDI). Neurology. 1994;44(5):837–42. 8190284 10.1212/wnl.44.5.837

[19] Mehta D, Cheema S, Davagnanam I, Matharu M. Diagnosis and treatment evaluation in patients with spontaneous intracranial hypotension. Front Neurol. 2023;14:1145949. 36970531 10.3389/fneur.2023.1145949 PMC10036855

[20] J‑p L, S‑d Z, F‑f H, M‑j L, X‑x M. The status of diagnosis and treatment to intracranial hypotension, including SIH. J Headache Pain. 2017;18(1):4. 28091819 10.1186/s10194-016-0708-8 PMC5236046

[21] Cheema S, Anderson J, Angus-Leppan H, Armstrong P, Butteriss D, Jones LC, Choi D, Chotai A, D’Antona L, Davagnanam I. Multidisciplinary consensus guideline for the diagnosis and management of spontaneous intracranial hypotension. J Neurol Neurosurg Psychiatry. 2023;94(10):835–43. 37147116 10.1136/jnnp-2023-331166 PMC10511987

[22] Kranz PG, Gray L, Amrhein TJ. Spontaneous Intracranial Hypotension: 10 Myths and Misperceptions. Headache. 2018;58(7):948–59. 29797515 10.1111/head.13328

[23] Volz F, Wolf K, Fung C, Carroll I, Lahmann C, Lützen N, Urbach H, Klingler JH, Beck J, El Rahal A. Impact of Spinal CSF Leaks on Quality of Life and Mental Health and Long-Term Reversal by Surgical Closure. Neurol Clin Pract. 2024;14(2):e200272. 38585435 10.1212/CPJ.0000000000200272 PMC10996905

[24] McEwen BS. Stress, adaptation, and disease. Allostasis and allostatic load. Ann N Y Acad Sci. 1998;840:33–44. 9629234 10.1111/j.1749-6632.1998.tb09546.x

[25] Liaw V, McCreary M, Friedman DI. Quality of Life in Patients With Confirmed and Suspected Spinal CSF Leaks. Neurology. 2023;101(23):e2411–e22. 37816637 10.1212/WNL.0000000000207763 PMC10752647

[26] Closs SJ, Staples V, Reid I, Bennett MI, Briggs M. The impact of neuropathic pain on relationships. J Adv Nurs. 2009;65(2):402–11. 19191938 10.1111/j.1365-2648.2008.04892.x

[27] Luetmer PH, Mokri B. Dynamic CT myelography: a technique for localizing high-flow spinal cerebrospinal fluid leaks. AJNR Am J Neuroradiol. 2003;24(8):1711–4. 13679297 PMC7973984

[28] Nadeau SE, Wu JK, Lawhern RA. Opioids and chronic pain: an analytic review of the clinical evidence. Front Pain Res. 2021;2:721357. 10.3389/fpain.2021.721357 PMC8915556 35295493

[29] Piechowiak EI, Aeschimann B, Häni L, Kaesmacher J, Mordasini P, Jesse CM, Schankin CJ, Raabe A, Schär RT, Gralla J. Epidural blood patching in spontaneous intracranial hypotension—do we really seal the leak? Clin Neuroradiol. 2023;33(1):211–8. 36028627 10.1007/s00062-022-01205-7 PMC10014648

[30] Farb RI, Nicholson PJ, Peng PW, Massicotte EM, Lay C, Krings T. Spontaneous Intracranial Hypotension: A Systematic Imaging Approach for CSF Leak Localization and Management Based on MRI and Digital Subtraction Myelography. Ajnr Am J Neuroradiol. 2019;40(4):745–53. 30923083 10.3174/ajnr.A6016 PMC7048504

